# Quantification of *Mycobacterium avium* subspecies in pig tissues by real-time quantitative PCR

**DOI:** 10.1186/1751-0147-55-26

**Published:** 2013-03-22

**Authors:** Taneli Tirkkonen, Timo Nieminen, Terhi Ali-Vehmas, Olli AT Peltoniemi, Gerard J Wellenberg, Jaakko Pakarinen

**Affiliations:** 1 Faculty of Veterinary Medicine, Department of Production Animal Medicine, University of Helsinki, POB 66, FIN 00014, Helsinki, Finland; 2 Present address of T. Tirkkonen: A-Farmers Ltd, POB 173, FIN 65101, Vaasa, Finland; 3 Animal Health Service (GD-Deventer), Arnsbergstraat 7, PO Box 9, Deventer, 7400 AA, The Netherlands; 4 Faculty of Agriculture and Forestry, Department of Applied Chemistry and Microbiology, University of Helsinki, Helsinki, Finland

**Keywords:** *Mycobacterium*, Pig, Porcine, Real-time qPCR, Quantification

## Abstract

**Background:**

Mycobacterioses in animals cause economical losses and certain *Mycobacterium avium* subspecies are regarded as potential zoonotic agents. The evaluation of the zoonotic risk caused by *M. avium* subspecies requires information about the quantities of *Mycobacterium* strains in infected animals. Because *M. avium* subspecies in pig tissues are difficult or even impossible to quantify by culturing, we tested the suitability of a culture-independent real-time quantitative PCR (qPCR) assay for this purpose.

**Methods:**

Mycobacterial DNA was extracted from porcine tissues by a novel method and quantified by *Mycobacterium* genus specific qPCR assay targeting the 16S rRNA gene.

**Results:**

The response of the qPCR assay to the amount of *M. avium* subspecies *avium* mixed with porcine liver was linear in the range of approximately log10^5^ to log10^7^*Mycobacterium* cells per 1 g of liver. The assay was validated with three other *M. avium* subspecies strains. When the assay was applied to porcine lymph nodes with or without visible lesions related to *Mycobacterium avium* subspecies infections, around 10^4^–10^7^ mycobacterial genomes per gram of lymph nodes were detected.

**Conclusions:**

The qPCR assay was found to be suitable for the quantification of *Mycobacterium avium* subspecies in porcine lymph nodes and liver.

## Background

*Mycobacterium avium complex* (MAC) is the most infectious group of environmental *Mycobacterium* strains being responsible for over 20% of human cases reported of having mycobacterial infections [1]. In total, 26.000 environmental *Mycobacterium* related infections were reported in 14 countries worldwide between 1991 and 1996 [1]. In Finland there were 12.2 infections per 100 000 inhabitants in 2012 [2]. Official international prevalence statistics are unavailable because these infections are not registered in most countries [3].

In addition to humans, environmental *Mycobacterium* strains infect poultry, pigs and ruminants in the food productions chains which may be a source of food borne illnesses in humans. Porcine mycobacteriosis is the most common animal mycobacterial disease in Finland, with long-term average prevalence of 0.34% and temporary peaks as high as 0.85% [4]. Mycobacteriosis has been diagnosed in pigs worldwide. *M. avium* subsp. *hominissuis* is a potential zoonotic pathogen that also infects pigs [5-7]. Infections of swine with *Mycobacterium avium* subspecies are typically associated to the lymph nodes in the neck and the gastrointestinal system [8]. The liver may be infected and sometimes also the spleen and lungs. Due to human mycobacterial infections, the European Union Legislation [9] regulates meat control practices in slaughterhouses. *Mycobacterium avium* subspecies infections in pigs are diagnosed presumptively in slaughterhouses by veterinary meat inspection officers. The lymph nodes and livers are examined visually at post-mortem inspection for granulomatous lesions. Meat and organs of infected animals may be graded as conditionally consumable after heat treatment depending on the country and the distribution of the lesions. These regulations cause economic losses to pig farmers and processing plants [3,8,10]. The visual examination is neither a specific nor sensitive method to detect mycobacteriosis in pigs. Specific methods for the typing of *M. avium* strains from Finland were earlier developed [6,7]. Alternative tests, potentially suitable for slaughterhouse use, have been developed, such as e.g. the detection of *Mycobacterium avium* subspecies antibodies in porcine blood samples [11]. However, verification of the diagnostic value of the serological tests requires quantification of *Mycobacterium avium* subspecies in porcine tissues. To assess the real human zoonotic risk it is essential to know the relation between porcine serological responses and the actual amount of *Mycobacterium avium* subspecies *avium* and *hominissuis* in the tissues. As far as we know no such research results exist from naturally infected pigs. Cultivation of *Mycobacterium* strains from animal samples and the final characterisation and determination of its phenotype has the disadvantage of taking a long time. Slowly growing *Mycobacterium* strains require between 3–6 weeks to form visible colonies on Lowenstein-Jensen media. Selective treatment required to kill background microbes inactivates also *Mycobacterium* strains [12]. Furthermore, the culture method may not reveal the exact concentration of *Mycobacterium* strains in a given sample [13,14].

In this study, we present a simple, rapid and accurate DNA extraction method that, in combination with a real-time qPCR method [15], can be used to quantify *Mycobacterium* strains in porcine tissue samples.

## Methods

### Bacterial strains

The following *M. avium* subspecies strains were used to validate the qPCR technique: *M. avium* subspecies *avium* ATCC 25291, *M. avium* subspecies *hominissuis* 9646/4 from Austria, *M. avium* subspecies *hominissuis* 9972/6 from Austria and *M. avium* subspecies *hominissuis* strain 2 from the Netherlands kindly provided by Gerard Wellenberg. These strains were identified as *M. avium* subspecies *hominissuis* as described by Wellenberg et al. [16]. The strains were cultivated on Middlebrook 7H11 agar with OADC enrichment.

### Quantification of *M. avium* subspecies by microscopy

For validation studies, *Mycobacterium* cells were collected from actively growing broth culture by centrifugation at 16.100 *g*. The cells were suspended in sterile water containing 1% peptone and 0.05% Tween-80. The cell density of the suspension was counted with a Bürker haemocytometer.

### Pig tissues

Eight lymph node samples (Table 1) were collected from slaughtered pigs infected with *M. avium* subspecies *hominissuis.* The sample collection and initial processing of samples was performed as described previously [16].

**Table 1:** **Quantification of *Mycobacterium *cells in lymph nodes originating from slaughter pigs suffering from a mycobacterial infection**

**Sample**	**Description**	qPCR cell equivalents* of mycobacterial DNA g-1 tissue
Pig 4, sample1.	Lesion	3 × 10^6^
Pig 4, sample 2.	outside the lesion part	below detection limit
Pig 9–5577, sample1.	Lesion	2 × 10^6^
Pig 9–5577, sample 2.	outside the lesion part	below detection limit
Pig Austria 3, sample 1.	Lesion	2 × 10^7^
Pig Austria 3, sample 2.	outside the lesion part	2 × 10^4^
Pig 187, sample 1.	Lesion	below detection limit
Pig 187, sample 2.	outside the lesion part	below detection limit

*Cell equivalent calculated based on the copy assumed number of one 16S rRNA gene per mycobacterial cell.

### DNA extraction

Mycobacterial DNA was isolated from tissue specimens (100 mg) or bacteria using the MagNA Pure LC DNA Isolation Kit III Bacteria & Fungi (Roche Diagnostics, Penzberg, Germany). The specimen was collected in a screw-capped 2 ml microcentrifuge tube mixed with 395 μl of Bacterial Lysis buffer and 35 μl of Proteinase K solution. However, the amount of isolated mycobacterial DNA was poor when the standard protocol was used. To increase the mycobacterial cell wall lysis the tissue was digested at 65°C under agitation at 160 rpm for 16 h. The tissue lysate was centrifuged at 16.100 *g* for 10 min to pellet *Mycobacterium* cells, and the supernatant was removed. The glass and silica granules in a BIO101 lysing matrix E tubes (Q Biogene, Irvine, CA, USA) were poured over the pellet and the pellet was homogenized two times in the FastPrep™ FP120 instrument (Bio101 Savant Instruments Inc., Holbrook, NY, USA) at 5.0 m s^-1^ for 40 s. The homogenate was centrifuged at 16.100 *g* for 10 min. DNA was isolated from the supernatant using the above mentioned kit and the KingFisher mL instrument (ThermoElectron, Helsinki, Finland).

### Real-time qPCR assay

A real-time qPCR method for the quantification of mycobacterial 16S rRNA genes was developed in former studies [15,17]. The 16S rRNA genes of mycobacteria were amplified using the genus-specific primers (MycoARB210 TTT GCG GTG TGG GAT GGG C, MycoARB585 CGA ACA ACG CGA CAA ACC A) and the products detected by the probes MycoFlu (CTC AGT CCC AGT GTG GCC GG, 3^′^ fluorescein-labelled) and MycoRed (CAC CCT CTC AGG CCG GCT AC, 5^′^ Red705-labelled, 3^′^ phosphorylated). Primers and probe specificity was confirmed with various target and non-target strains [10]. Each run included control/experimental samples, a reagent control (reagents used to extract DNA to rule out experimental contamination during DNA extraction), a negative reagent control (DNA free water) and a positive control (standard curve with known amounts of *Mycobacterium lentiflavum*).

### Spiking of *Mycobacterium* cells to pig liver

To validate the qPCR method for the quantification of *M. avium* subspecies we extracted DNA from pure cultures of four *M. avium* subspecies strains and from porcine tissue samples that were healthy or spiked with *M. avium* subspecies. Healthy pig liver (0.1 g) was spiked with five parallel 10-fold dilutions of *M. avium* subspecies *avium* ATCC 25291 cells (1 × 10^4^ to 1 × 10^7^ cells per g), quantified by microscopic counting.

## Results

### Quantification of *M. avium* subspecies by microscopy and qPCR

We tested the response of the *Mycobacterium* genus specific qPCR assay on four *M. avium* subspecies strains. The results obtained by the qPCR assay correlated closely with the microscopic counts of the four *M. avium* subspecies strains tested (Table 2). The maximum difference between the qPCR and microscopy counts was less than one 10log unit.

**Table 2:** **Quantification of *Mycobacterium *cells by microscopy and by qPCR**

**Strain/sample**	**Microscopy cells ml**^**-1**^	qPCR cell equivalents* of mycobacterial DNA ml^**-1**^
*M. avium* ATCC 25291	1 × 10^6^	2 × 10^6^ (n = 2)
*M. avium* ATCC 25291	1 × 10^8^	6 × 10^7^ (n = 2)
*M. avium* Austria 9646/4	5 × 10^8^	2 × 10^8^
*M. avium* Austria 9972/6	7 × 10^9^	3 × 10^9^
*M. avium* Netherlands 2	2 × 10^9^	9 × 10^8^
*M. avium* ATCC 25291 + 0.1 g of liver	1 × 10^4^	7 × 10^3^
*M. avium* ATCC 25291 + 0.1 g of liver	1 × 10^6^	7 × 10^5^

*Cell equivalent calculated based on the assumed single copy number of 16S rRNA as information regarding the exact number of 16S rRNA in *M. avium* subspecies *hominissuis* is not available so far.

We were also able to quantify *M. avium* subspecies *avium* ATCC 25291 DNA when bacteria were mixed with porcine liver tissue (Table 2).

### Response of qPCR assay to *M. avium* subspecies spiked in liver

**Figure 1:**
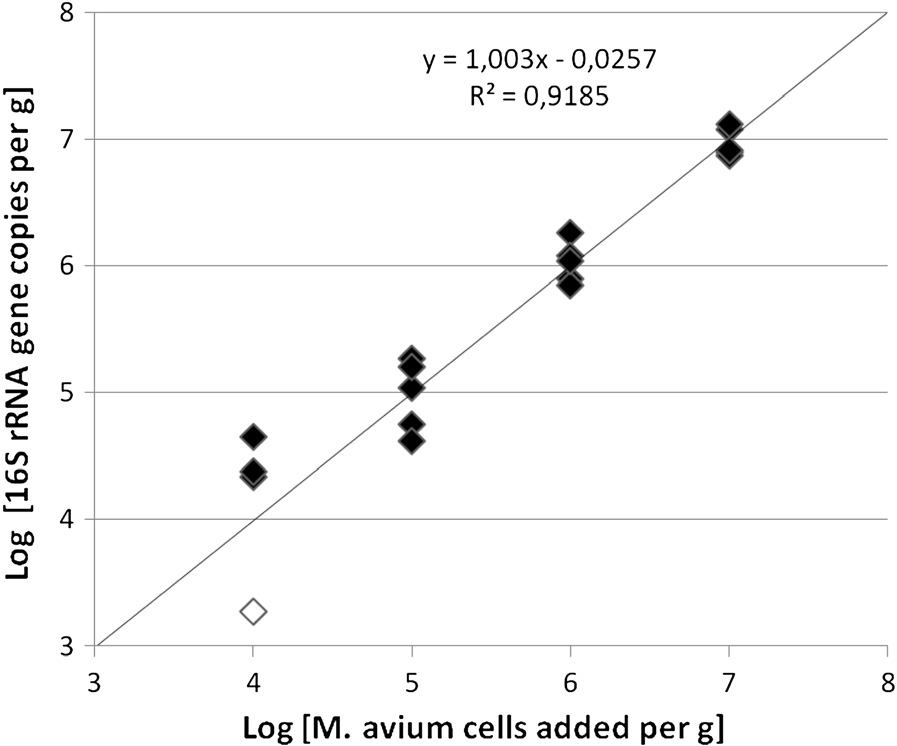
**Response of the *Mycobacterium*-specific qPCR assay to *M. avium *subspecies *avium *ATCC 25291 cells in pig liver.** Mycobacterial DNA was quantified in five parallel extracts of pig liver spiked with *M. avium* subspecies *avium* (1 × 10^4^ to 1 × 10^7^ bacteria per gram). Open symbols denote qPCR results below the detection limit (4 × 10^4^ mycobacteria per gram).

To test the detection limit of the qPCR in porcine tissue, we analyzed specimens (0.1 g) of healthy pig liver spiked with five 10-fold decimal dilutions of *M. avium* subspecies *avium* ATCC 25291 cells (1 × 10^4^ to 1 × 10^7^ bacteria per gram). Each dilution was extracted and measured as five parallels. Figure 1 shows the results of the qPCR analysis of the liver spiked with *M. avium* subspecies *avium*.

The response of the qPCR assay to the logarithmic amount of *M. avium* subspecies *avium* added to pig liver was linear approximately in the range of log10^5^ to log10^7^ bacteria per gram. Three out of the five parallel specimens spiked with 10^4^*M. avium* subspecies *avium* per gram were also detected but were out of the linear range.

### Quantification of *Mycobacterium* cells from infected lymph nodes by the qPCR assay

*Mycobacterium* cells were quantified in the lymph node tissues from four infected pigs. Cell equivalents of mycobacterial DNA between 2 × 10^4^ to 2 × 10^7^ were detected per gram of tissue using the qPCR assay (Table 1). Each affected tissue was sampled, both within the lesions and adjacent to the lesions, to study the distribution of *Mycobacterium* cells in the affected organs. In three out of the four cases studied, the concentration of *Mycobacterium* cells was at least 1000 times higher in the lesion part than in the adjacent part of the affected tissue.

## Discussion

*Mycobacterium* strains may cause serious infections in animals and humans. Large economic losses are caused by many mycobacterial species. A high risk of transmission of infection from animal to human exists. The knowledge of the exact pathogen concentration in mycobacterial contaminated pork might be an important parameter to give information on the infection risk for humans. A low mycobacterial porcine tissue content may be indicative for an environmental contamination. A high concentration of *Mycobacterium* cells in porcine tissue may indicate a higher risk for humans to become infected after consumption of infected pork and may therefore represent a higher zoonotic risk. Earlier authors have described qPCR and serological results based on experimental *M. avium* subspecies porcine infections [11,18]. Miranda et al. [19] found *M. avium* subsp. *paratuberculosis* in four out of fifty examined tissue samples with PCR whereas Klanicova et al. [20] examined various purchased meat products for *M. avium* subspecies by qPCR. However to the best of our knowledge this is the first publication to report the quantification of mycobacterial content in naturally infected porcine tissue by qPCR and verifying it with another quantification method.

The problems connected to cultivation have increased the interest in culture-independent methods. Different microscopical methods have been applied to detect mycobacterial cells in animal and environmental originating samples [21]. The tendency of mycobacterial cells to clump may hinder accurate microscopic as well as cultivation based quantification of *Mycobacterium* cells. The specificity and sensitivity of these cultivation/microscopy methods need to be significantly improved before they can be applied to the analysis and quantification of *Mycobacterium* cells from animal samples.

Therefore, new methods, such as quantitative PCR methods in combination with reliable DNA extraction methods, are required. In general, a variety of methods can be used for DNA isolation from animal samples, from boiling the sample in distilled water, autoclaving, disruption by glass beads or sonication, to the use of different enzymes and surfactants. However, isolation of nucleic acids from *Mycobacterium* cells is more difficult than from other microorganisms because of the thick peptidoglycan layer characteristic to the mycobacterial cell wall, which makes it resistant to a number of lysis buffers. Moreover, certain samples of animal origin may contain various inhibitors of PCR amplification [22].

A number of published protocols and commercial kits are available for the extraction of DNA from mycobacterial isolates. However, most of these cannot be used to the determination of mycobacterial DNA from porcine tissues. Commercial kits show an excellent correlation with 16S rDNA sequencing results representing rapid, specific and versatile species identification of the most prevalent NTM-species from cultures [23]. Recently several novel qPCR methods have been developed for the detection of *Mycobacterium* strains from human, animal and environmental originating samples [10,18]. However, less laborous and complex methods are needed.

Our DNA extraction and real-time quantitative PCR protocol is a simple and effective method for the detection and quantification of *Mycobacterium* strains in porcine tissues. The DNA extraction method was found to be efficient in extracting different amounts of *M. avium* subspecies spiked into healthy pig liver and is also suitable to detect *M. avium* subspecies in porcine tissue samples. The qPCR method was shown to provide reliable quantitative results when *M. avium* concentrations ranged from log10^5^ to log10^7^ (Figure 1). These tissue concentrations can be regarded as relatively high, but to the best of our knowledge no exact information regarding the smallest zoonotic infection dose for *M. avium* subspecies is available. The total mycobacterial count using the developed extraction method for tissue lesions was as high as 10^7^ cells per gram, indicating an infection.

## Conclusions

Our protocol provides a novel, efficient and simple strategy to improve the performance of qPCR with excess of animal DNA in the background. The improved protocol may enable the detection of total mycobacterial cells also from samples without visible lesions.

## Competing interests

The authors declare that they have no competing interests.

## Authors’ contributions

TT, TA-V, JP and GW participated in the discussion on the study design, collection of the samples and carried out the analysis. TT, TA-V, JP, TN and OP participated interpretation of the data. TA-V, TN, JP, GW and OP helped to draft the manuscript. TT wrote the final manuscript. All authors read and approved the final manuscript.
